# Improving quality of breast cancer surgery through development of a national breast cancer surgical outcomes (BRCASO) research database

**DOI:** 10.1186/1471-2407-12-136

**Published:** 2012-04-03

**Authors:** Erin J Aiello Bowles, Heather Spencer Feigelson, Tom Barney, Katherine Broecker, Andrew Sterrett, Kimberly Bischoff, Jessica Engel, Gabrielle Gundersen, Johanna Sheehey-Jones, Richard Single, Adedayo Onitilo, Ted A James, Laurence E McCahill

**Affiliations:** 1 Group Health Research Institute, Group Health Cooperative, 1730 Minor Ave, Suite 1600, Seattle, WA 98101, USA; 2 Institute for Health Research, Kaiser Permanente Colorado, 10065 E Harvard Ave, Suite 300, Denver, CO 80237, USA; 3 Van Andel Research Institute, 333 Bostwick Ave NE, Grand Rapids, MI 49503, USA; 4 Lacks Cancer Center, Saint Mary's Health Care, 250 Cherry St, Grand Rapids, MI 49503, USA; 5 Marshfield Clinic Research Foundation, Marshfield Clinic, 1000 North Oak Ave, Weston, WI 54449, USA; 6 Fletcher Allen Health Care, Colchester Avenue, Burlington, VT 05401, USA; 7 College of Medicine, University of Vermont, 89 Beaumont Ave, Burlington, VT 05405, USA

## Abstract

**Background:**

Common measures of surgical quality are 30-day morbidity and mortality, which poorly describe breast cancer surgical quality with extremely low morbidity and mortality rates. Several national quality programs have collected additional surgical quality measures; however, program participation is voluntary and results may not be generalizable to all surgeons. We developed the Breast Cancer Surgical Outcomes (BRCASO) database to capture meaningful breast cancer surgical quality measures among a non-voluntary sample, and study variation in these measures across providers, facilities, and health plans. This paper describes our study protocol, data collection methods, and summarizes the strengths and limitations of these data.

**Methods:**

We included 4524 women ≥18 years diagnosed with breast cancer between 2003-2008. All women with initial breast cancer surgery performed by a surgeon employed at the University of Vermont or three Cancer Research Network (CRN) health plans were eligible for inclusion. From the CRN institutions, we collected electronic administrative data including tumor registry information, Current Procedure Terminology codes for breast cancer surgeries, surgeons, surgical facilities, and patient demographics. We supplemented electronic data with medical record abstraction to collect additional pathology and surgery detail. All data were manually abstracted at the University of Vermont.

**Results:**

The CRN institutions pre-filled 30% (22 out of 72) of elements using electronic data. The remaining elements, including detailed pathology margin status and breast and lymph node surgeries, required chart abstraction. The mean age was 61 years (range 20-98 years); 70% of women were diagnosed with invasive ductal carcinoma, 20% with ductal carcinoma in situ, and 10% with invasive lobular carcinoma.

**Conclusions:**

The BRCASO database is one of the largest, multi-site research resources of meaningful breast cancer surgical quality data in the United States. Assembling data from electronic administrative databases and manual chart review balanced efficiency with high-quality, unbiased data collection. Using the BRCASO database, we will evaluate surgical quality measures including mastectomy rates, positive margin rates, and partial mastectomy re-excision rates among a diverse, non-voluntary population of patients, providers, and facilities.

## Introduction

The Institute of Medicine report "Crossing the Quality Chasm", emphasized high quality care should be safe, timely, effective, efficient, equitable, and patient-centered [1]. The common measures of surgical quality are 30-day morbidity and mortality, which are not ideal metrics for breast cancer procedures that have extremely low morbidity and < 0.5% mortality. These measures only reflect outcomes of surgery, but do not consider the surgical process or institutional structure - other aspects that can influence the overall procedure quality [2]. Several experts have agreed that the quality of cancer surgery is currently the "black box" of cancer care, subject to wide variability at provider-, facility-, and regional-levels, emphasizing the need for new standard, quality measures [3].

The University of Vermont Breast Cancer Surgical Outcomes (VBCSO) database was developed in 2003 to establish the feasibility of measuring potential breast cancer surgical quality indicators [4]. The VBCSO database included 910 women diagnosed with breast cancer between April 1, 2003- March 30, 2008 who underwent initial breast cancer surgery at a single institution. Trained nurses abstracted medical record data on initial mastectomy rate, close and positive margin rates, number of surgeries, number of lymph nodes obtained during sentinel node biopsy and/or axillary dissection, use of intraoperative pathology assessment for sentinel nodes, and days between diagnosis and initial surgery. Subsequently, these data have demonstrated variation in surgical outcomes among surgeons who practice at one institution [4].

We extended the VBCSO database to three Cancer Research Network (CRN) health plans to develop a large, non-voluntary, multi-institution database to study breast cancer surgical quality. The CRN is a consortium of 14 non-profit research centers based in integrated healthcare delivery organizations within the HMO Research Network [5]. The purpose of this paper is to describe the combined Breast Cancer Surgical Outcomes (BRCASO) research database, structure, data elements, and study methods in detail, including important lessons learned in collecting surgical outcomes data. We plan to use these data to evaluate variation in surgical quality measures (initial mastectomy rate, initial partial mastectomy positive margin rate, and initial partial mastectomy re-excision rate) by patient-, surgeon-, facility-, and region-level factors.

## Methods

Data from the VBCSO database were combined with data from three CRN health plans (Group Health Cooperative, Kaiser Permanente Colorado, and Marshfield Clinic) to form a single, large, multi-site database called BRCASO, which resides at the Van Andel Research Institute (VARI). All study procedures were approved by Institutional Review Boards at the University of Vermont, VARI, and Kaiser Permanente Colorado (through cooperative review for the CRN health plans); we received a waiver of consent to collect patient and provider-level data. The waiver was granted based on the fact that this study was a retrospective review of standard treatment data and of minimal risk to the patients; all identifiable information was kept at local sites, and only a deidentified data set was shared for analysis and reporting.

### Study population

A total of 6095 women ≥18 years old were diagnosed with stage 0-IV breast cancer between January 1, 2003 and December 31, 2008. To be eligible for inclusion in the database, women had to have their initial breast cancer surgery performed by a surgeon employed by the University of Vermont or one of the CRN health plans (including CRN health plan-contracted facilities). We included all women with ductal carcinoma in situ (DCIS), invasive ductal (IDC) or lobular carcinoma (ILC); all other breast pathologies were excluded. We also excluded males, women with initial surgery at an external facility by a non-CRN health plan-employed surgeon, women with breast cancer diagnosed on prophylactic mastectomy, and women with breast cancer diagnosed but never treated surgically.

### BRCASO database

**Figure 1:**
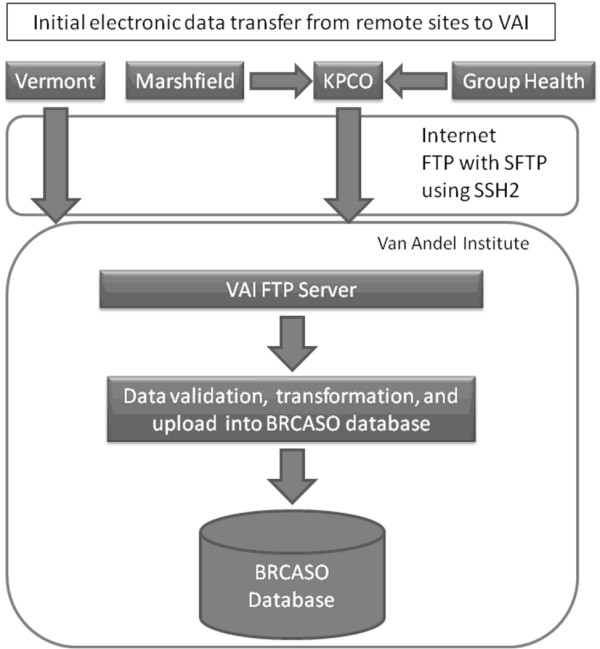
**Shows how electronic data were transferred from each Cancer Research Network site (Marshfield and Group Health) to KPCO (Kaiser Permanente Colorado) before transfer to VARI (Van Andel Research Institute)**. Vermont transferred data directly to VARI. Data were routed through the VARI FTP (File Transfer Protocol) server and underwent initial validation before transfer to the BRCASO database.

**Figure 2:**
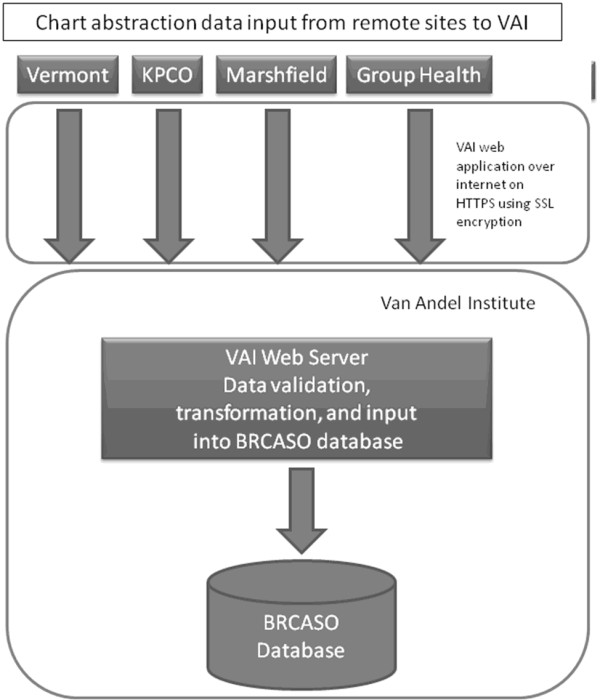
**Shows how chart abstraction data were collected from each Cancer Research Network site (KPCO [Kaiser Permanente Colorado], Marshfield, and Group Health) and Vermont and entered into a secure (HTTPS) online web application hosted by VARI**. Data underwent initial validation before transfer to the BRCASO database.

The BRCASO database is stored on a SQL server 2008 database at VARI, the primary site for this research. Because of the multi-site nature of this study, we followed strict security procedures to ensure confidentiality of data transfer to VARI. Figure 1 shows electronic limited data sets from each CRN institution were collated at a single site (Kaiser Permanente Colorado), before transfer to VARI through a Secure File Transfer Protocol (SFTP) site using Secure Shell (SSH2) encryption. The University of Vermont transferred electronic data directly to VARI using the same SFTP site. Figure 2 shows chart data were abstracted at individual institutions and entered into a secure (HTTPS) online web application hosted by VARI. VARI stored and collated all electronic and chart abstracted data behind their firewall, where they were only accessible to programmers and system administrators at VARI.

### Electronic administrative data

Each CRN health plan collects electronic administrative data on inpatient and outpatient procedures, health plan enrollment, pharmacy dispensings, laboratory tests, and other elements as part of clinical care. Each health plan also links to their local Surveillance, Epidemiology and End Results (SEER) or state cancer registry. All electronic data elements for CRN sites are part of the Virtual Data Warehouse (VDW) where they are formatted in a standard fashion across sites, but the data themselves are housed at each individual site [5]. For BRCASO, a programmer at one site wrote programs to identify eligible breast cancer cases and extract relevant administrative data that could be run against the VDW at each site. We extracted electronic information from: tumor registry data on diagnosis date, diagnosis type, laterality, estrogen and progesterone receptor status, and tumor grade; inpatient and outpatient Current Procedure Terminology (CPT-4) codes including date and type of procedures (partial mastectomies, total mastectomies, and lymph node surgeries) with de-identified surgeon and facility IDs; and demographic data on age, height, weight, geocoded income and education, ethnicity, race, and insurance type. These data were used to prefill the BRCASO database to reduce the amount of information that needed to be collected manually from the medical records.

### Abstracted medical record data

Each site reviewed their own electronic medical records for women eligible for the study. All abstractors had previous experience abstracting cancer patients' medical records. We developed a detailed data manual to standardize abstracted information (Additional file 1). This comprehensive manual provided not only data definitions, but also preferences for which clinical report information was generally most accurate and an overall context of how the data element would be used in this study. Chart abstraction was piloted at each institution using paper forms and results for 10 cases from each site were discussed via teleconference before finalizing the manual and database. All electronic pre-filled data were viewable within the database, and could be edited if discrepancies were noted between administrative and chart data. Each patient was automatically marked as complete once every data field was complete, and the record was then routed to a SQL database at VARI.

### Clinical details abstracted from medical records included

• Surgical breast procedures (multiple records per person allowed): indication (known malignancy vs. undiagnosed breast lesion), anesthesia type, tumor localization technique, intra-operative margin assessment, surgeon specimen orientation technique, breast reconstruction

• Surgical margins (collected per procedure): direction and distance of closest uninvolved margins by invasive carcinoma and/or DCIS, direction of margins involved (i.e. positive margin) by invasive carcinoma and/or DCIS

• Tumor information: tumor sizes (pathologic and as estimated by imaging prior to first treatment), size of invasive and DCIS components, presence of residual tumor in first and subsequent surgical specimens, synchronous tumors, final pathological diagnosis, lymphovascular invasion, perineural invasion, HER2 result, prophylactic mastectomy pathology

• Lymph nodes: type of procedure (sentinel lymph node [SLN] biopsy, axillary sampling/dissection), intra-operative SLN evaluation, SLN histology, SLN and axillary positive and total node counts

• Other: Neoadjuvant treatment, imaging performed, known multi-focal and multi-centric status pre-operatively, prior breast cancer history

### Quality control

Each chart abstractor was trained by the lead chart abstractor at the University of Vermont. Regular abstractor meetings occurred throughout the funding period to address specific questions. Chart abstractors could also post questions to a private SharePoint site that were answered by the lead chart abstractor or principal investigator and then disseminated to all abstractors for review. After completing 1,000 charts, we reviewed frequencies of BRCASO data elements by institution to check for inconsistencies, missing data, or distributions differences by institution. A report was provided back to each site, where abstractors confirmed any inconsistencies and made changes to the BRCASO database if needed. We continued to review frequencies, perform variable cross-checks, and clean data as necessary throughout the abstraction period. The database was designed to keep a detailed log of all entries and edits to the data by user ID to maintain data integrity.

## Results

We have complete data on 4524 of 6095 women (74.2%) and their surgeries in the BRCASO database, to date. The mean age was 61 years (range 20-98 years) and 94% of the study population was white. Approximately 40% of the study population were insured by Medicare and 4% by Medicaid. Nearly 70% of women were diagnosed with IDC, 20% with DCIS, and 10% with ILC.

By using electronic administrative data, CRN health plans pre-filled 22 of 72 (30%) data fields. The remainder of the 50 fields required abstraction from the medical record. In addition, several electronic data fields occasionally required editing by the chart abstractor to update and clarify diagnosis and procedure dates, performing surgeon, surgical facility, and lymph node procedures. The total time for chart abstraction ranged from 30-90 minutes per chart depending on the number of surgeries per woman, and the availability of pathology and operative reports within the medical record. A list of potential quality measures that will be evaluated using these data for variation across women, surgeons, and facilities are in Table 1. We specifically selected eight measures that we felt would be meaningful to patients and were previously evaluated in the VBCSO database; [4] we included two additional measures (use of needle biopsy for pre-operative diagnosis, and use of neoadjuvant chemotherapy for T2/T3 tumors) that showed broad variation during data collection. In general, these were regularly recorded and easily found in automated data or medical records, which increases the adaptability of our methods to other institutions.

**Table 1:** Potential surgical quality measures to be evaluated using BRCASO data

	SURGICAL OUTCOME MEASURE
**PRIMARY TUMOR**	Mastectomy rate

	Positive/close margin rate following initial partial mastectomy

	Partial mastectomy re-excision rate

	Use of needle biopsy for pre-operative diagnosis

	Use of neoadjuvant chemotherapy for T2/T3 tumors

**AXILLARY MANAGEMENT**	Number of sentinel nodes identified

	Intra-operative lymph node assessment

	Nodes examined following axillary dissection

	Completion axillary dissection rate following positive sentinel lymph node

**HEALTH CARE DELIVERY**	Initial breast cancer surgery within 30 days

## Discussion

Developing quality measures for breast cancer surgery is critical given the high frequency of surgical breast procedures and potential variation that exists across facilities and surgeons. Wasif et al. comment that quality measures must 1) be acceptable to various stakeholders; 2) include measureable elements; and 3) impact outcomes [2]. While several national and international groups have developed quality cancer care measures, most focus on treatment other than breast surgery and participation in these programs tends to be voluntary. BRCASO aims to develop a more comprehensive, validated set of breast cancer surgical quality measures, and use these data to conduct research on surgical quality variation among a generalizable (i.e. non-voluntary) population.

Current quality measurement programs for cancer care delivery, while valuable, are limited in their ability to specifically address surgical quality and provide detailed data for research. Two of these programs, QOPI - ASCO's Quality Oncology Practice Initiative [6] and the American College of Surgeons' (ACoS) Commission on Cancer (CoC), [7] are primarily geared toward measuring and documenting overall quality of cancer care. Together, the CoC and American Cancer Society developed the National Cancer Data Base, one of the largest national registries of new cancer diagnoses. The data collected for each of these efforts primarily relate to documentation and treatment other than surgery.

One recent program dedicated primarily to breast care quality improvement is the American Society of Breast Surgeons' Mastery of Breast Surgery Program, another voluntary initiative that helps surgeons document their clinical performance; early results showed program participation rates among surgeons practicing in academic settings was low [8]. Facilities may also receive certification from the National Accreditation Program for Breast Centers (NAPBC) [9]. Accredited breast centers are required to meet certain surgical standards including > 50% of early stage breast cancer patients treated with breast conserving surgery, SNL for all patients with stage I or II disease, and diagnosis with palpation- or image-guided biopsy rather than open biopsy.

Perhaps the most detailed surgical quality measures come from the National Consortium of Breast Centers, Inc. and its National Quality Measures for Breast Centers (NQMBC) program [10]. The NQMBC's quality measures include 37 measures for breast cancer diagnosis and treatment including surgical timeliness, pathology timeliness, pathology report completeness (tumor size, margins, lymph nodes, and specimen sampling adequacy), sentinel node biopsy, breast conservation surgery rate, and re-excision rate. The program includes over 200 voluntary participants and allows them to compare their performance with other centers across the US. Members can collect all measures or a select few depending on their certification level. The validity of some of these measures as reasonable quality measures has not been tested in a broad population of patients.

The data collected from the BRCASO study will enable us to evaluate a number of quality measures specific to breast cancer surgery. Our three initial proposed surgical quality measures are 1) initial mastectomy rate, 2) initial partial mastectomy positive margin rate, and 3) initial partial mastectomy re-excision rate. At this time, we are not recommending benchmarks for these measures, but instead, will evaluate variability in these measures by patient-, surgeon-, facility-, and regional-level factors, and by invasive and in situ diagnoses that may inform future benchmarks. We will be able to control these outcomes for clinical factors such as estimated tumor size, known multi-centric disease, breast imaging, anesthesia, localization technique, neo-adjuvant treatment, and detailed tumor characteristics. We also have detailed data on timeliness of procedures, extent of lymph node procedures, positive node counts, and reconstruction, which may serve as additional surgical quality measures. Most importantly, we have collected margin status for each procedure performed, which is important in assessing surgical quality and cannot be extracted from tumor registry data.

The BRCASO database has the potential to be one of the largest, multi-site breast cancer surgical quality research databases to-date. The research possibilities may extend even beyond surgical quality measures. One of the major differences between BRCASO and the national efforts described above is that BRCASO does not rely on voluntary reporting. BRCASO includes a diverse geographic sample of all surgeons who practice at our varied institutions (health maintenance organization, university hospital, contracted community hospitals). In addition, a proportion of the data from the CRN sites were collected from electronic administrative data, a process that has the potential to be extended to other organizations with similarly organized billing data.

However, the BRCASO database is not without limitations. Our electronic administrative data were less sufficient than we had hoped for in pre-filling the database. Thus our chart abstraction took longer than we anticipated, and we fell short of the originally anticipated 6,095 cases. We have no detailed information on surgeon training, surgeon specialty, patient breast size, family history or patient preferences - all of which can account for variability in surgical procedures and quality. The focus of BRCASO was to collect data on specific short-term surgical outcomes such as reexcisions rates; thus we do not have data on long-term outcomes of recurrence or mortality, but plan to expand our efforts to collect these data in the future.

We learned several important lessons that others may want to consider when collecting surgical quality data. First, while electronic administrative data were useful in prefilling some of our data elements, they have serious limitations. Most importantly, there is no way to get accurate detailed margin or lymph node data from electronic administrative data; re-excision data may also be inaccurate as repeat procedures do not appear to be well documented in electronic data. Tumor registry data generally represent final surgical procedure and final pathology, but do not offer a clear characterization of multiple procedures that a patient may receive. The CoC database does collect information on positive/negative margin status, but does not include detail on margin distance or direction. Until more detailed, standard definitions are developed and implemented, we can only obtain detailed surgical quality data through medical record abstraction; but these data are time consuming and expensive to collect. We noted modest variation in the information provided in pathology, surgical operative, and other clinical reports between organizations and facilities. While standardizing these reports across institutions would be ideal, it was not possible in this retrospective study. Therefore, having highly trained medical record abstractors, a detailed coding manual, and regular channels for abstraction-related queries were invaluable to reducing data discrepancies in our studies.

Collecting a large amount of multi-site surgical quality data is a complex task - but the potential benefits and knowledge gained may be substantial. While some of the national efforts mentioned here will provide larger databases with which to evaluate quality of breast cancer care, only one (the NQMBC program) appears to collect the level of detail needed to evaluate quality of surgical care. These databases also rely solely on self-reported information from providers who opt-in to each system, and may be more likely to involve high volume breast surgeons. These providers may not be representative of the national population of surgeons because many surgeons performing breast surgery in the United States are neither high volume breast surgeons nor work within a certified breast center [8]. Our study collected data on every surgeon who performs breast procedures at one of four geographically disparate institutions, with variable volume in breast surgery. Standardizing clinical data collection and reporting efforts across programs and databases (including ours), with institutional participation rather than voluntary, and publicly reporting results will likely be necessary to develop timely benchmarks for surgical quality. Additional programming work, such as Natural Language Processing, may help improve data collection timeliness and efficiency by automatically interpreting text from electronic medical records, thus reducing the need for manual abstraction. Creating standard templates in electronic medical records for pathology and surgery reports may be another method that can improve both quality and consistency of data collected and reported. We hope the work from BRCASO provides a stepping stone for research on and development of standard surgical quality measures that can be generalized to and implemented by other providers and institutions.

## Competing interests

The authors declare that they have no competing interests.

## Authors' contributions

EJAB, HSF, RS, AO, TAJ, and LEM conceived of the study, and participated in its design and coordination and helped to draft the manuscript. TB and AS developed the electronic database for data collection, prefilled all electronic administrative data, and helped draft the manuscript. KaB, KiB, JE, GG, and JSJ developed study materials, including the chart abstraction manual, trained and provided support to abstractors, and helped draft the manuscript. All authors have read and approved the final manuscript.

## Pre-publication history

The pre-publication history for this paper can be accessed here:

http://www.biomedcentral.com/1471-2407/12/136/prepub

## Supplementary Material

Additional file 1 **BRCASO chart abstraction manual**. Detailed chart abstraction methods and protocol used in the BRCASO study. Click here for file
