# Supplementary material for: Improving quality of breast cancer surgery through development of a national breast cancer surgical outcomes (BRCASO) research database
Source: BMC Cancer. 2012 Apr 3;12:136. doi: 10.1186/1471-2407-12-136 (PMC3350402; doi:10.1186/1471-2407-12-136)
Supplement: Additional file 1 — BRCASO chart abstraction manual. Detailed chart abstraction methods and protocol used in the BRCASO study. [file 1471-2407-12-136-S1.PDF]

Instructions for Completing Breast Cancer Surgery Outcomes (BRCASO) Data Form

General Instructions: Please select single criteria or answer when single select option is offered. If you have some concern about the best answer, please do not select two choices (for example: circle positive and also circle negative with a comment). Database will only accept a single answer, unless specifically indicated as multi-select. There is no comment field available.

Patient Eligibility Criteria:

Eligible patients should meet each of the following criteria:

1 Female patients diagnosed with breast cancer

and

2 Underwent initial breast cancer or axillary surgical procedure and subsequent cancer-directed breast or axillary procedures related to the incident of breast cancer (diagnostic or cancer-directed) between 01/01/2003 and 12/31/2008 at your institution or a facility under contract by your organization to provide care where the pathology report demonstrated cancer

and

3 Patient underwent initial breast cancer or axillary surgical procedure (diagnostic or cancer-directed) at your institution between 01/01/2003 and 12/31/2008

OR

3. Patient underwent initial breast cancer or axillary surgical procedure (diagnostic or cancer-directed) between 01/01/2003 and 12/31/2008 at a facility under contract by your organization to provide care **AND** by surgeon for initial and subsequent procedures was employed by your institution

Examples of Eligible Patients

1) Patients with confirmed Breast Cancer diagnosis prior to cancer directed surgery:
   a) Diagnosed by fine needle aspirate (FNA)
   b) Diagnosed by core biopsy
   c) Surgical excisional biopsy
2) Patients with concerning or suspicious breast masses or radiographic lesions undergoing initial surgical excision (surgical "breast biopsy")
   a) Patients with a Cat 4, 5 mammogram/MRI/Ultrasound who undergo initial excisional biopsy or initial partial mastectomy or initial total mastectomy
   b) Patient with a palpable breast mass where surgeon performs a "partial mastectomy" or breast biopsy and the pathology report identifies breast cancer
3) Breast Cancer Clinical Stages: 0, I, II, III, IV

Final Pathology should demonstrate one of the following:

1) Invasive Ductal Cancer
   a) 82113 Tubular adenocarcinoma
   b) 84803 Mucinous adenocarcinoma
   c) 80503 Papillary Carcinoma, NOS
   d) 85103 Medullary Carcinoma
   e) 85003 Infiltrating ductal carcinoma, NOS
   f) 85213 Infiltrating ductal carcinoma
   g) 85223 Infiltrating ductal and lobular carcinoma
   h) 85233 Infiltrating ductal mixed w/ other types of carcinoma
   i) 85303 Inflammatory carcinoma
   j) 85413 Paget disease & infiltrating ductal carcinoma of breast
2) Invasive Lobular Cancer
   a) 85203 Lobular carcinoma, NOS
   b) 85243 Infiltrating lobular mixed w/ other types of carcinoma
3) Ductal Carcinoma In Situ (DCIS)
   a) 82012 Cribriform carcinoma in situ
   b) 82302 Ductal carcinoma in situ, solid type
   c) 85002 Intraductal carcinoma, noninfiltrating, NOS
   d) 85222 Intraductal carcinoma & lobular in situ
   e) 85232 DCIS Cribriform/micropapillary
   f) 85432 Paget disease & intraductal carcinoma
   g) 85433 Paget disease & intraductal carcinoma of breast

Excluded patients

a) Male Patients
b) Patients who undergo initial breast surgery (either excisional biopsy or initial cancer-directed surgery) at an outside treatment facility by a different surgeon and undergo completion cancer-directed breast or axillary surgery at your institution by one of your surgeons (two surgeon problem)
c) Patients who undergo initial breast surgery (either excisional biopsy or initial cancer-directed surgery) at your facility and undergo completion of cancer- directed breast or axillary surgery at a second institution by another surgeon (two surgeon problem)
d) Breast Cancer diagnosed on prophylactic mastectomy e)

Breast Cancer diagnosed but never treated surgically

f) Patients undergoing breast/axillary/chest wall operations performed solely for a recurrence of previous breast cancer (defined as breast cancer > 6 months previously <u>and</u> having previous breast cancer operation).
g) Patients who underwent > 6 months of endocrine therapy before surgery where intent was to avoid surgery all together in elderly or infirm patients.
h) Excluded pathology codes:

80003	Neoplasm, malignant
80102	Carcinoma in situ, NOS
80103	Carcinoma, NOS
80323	Spindle cell carcinoma, NOS
80413	Small cell carcinoma, NOS
80502	Papillary carcinoma in situ
80703	Squamous cell carcinoma, NOS
80713	Squamous cell carcinoma, keratinizing, NOS
81402	Adenocarcinoma in situ, NOS
81403	Adenocarcinoma, NOS
82002	Adenoid cystic carcinoma in situ

82003 Adenoid cystic carcinoma

82013	Cribriform carcinoma, NOS
82303	Solid Carcinoma, NOS
82463	Neuroendocrine carcinoma, NOS
82603	Papillary adenocarcinoma, NOS
84012	Apocrine DCIS
84013	Apocrine adenocarcinoma
85012	Comedocarcinoma, noninfiltrating
85013	Comedocarcinoma, NOS
85023	Secretory Carcinoma of breast
85032	Noninfiltrating intraductal papillary adenoca
85033	Intraductal papillary adenoca w/ invasion
85042	Noninfiltrating intracystic carcinoma
85043	Intracystic carcinoma, NOS
85072	Intraductal micropapillary carcinoma
85073	Invasive micropapillary carcinoma
85133	Atypical Medullary carcinoma
85202	Lobular carcinoma in situ, NOS
85402	Paget disease
85403	Paget disease, mammary
85733	Adenoca with apocrine metaplasia
85753	Metaplastic carcinoma, NOS
88023	Giant cell sarcoma (except of bone)
88103	Fibrosarcoma, NOS
89833	Malig Adenomyoepithelioma w/o invasion
90203	Phyllodes tumor, malignant
91203	Hemangiosarcoma
91803	Osteosarcoma, NOS
96843	Malignant Lymphoma
96913	Follicular Lymphoma
	Atypical ductal hyperplasia
	Atypical lobular hyperplasia

Documents needed for auditing:

Initial surgeon consult

Initial screening or diagnostic mammogram report

Additional view mammogram reports

Breast Ultrasound report

Breast MRI report

CT report

Pathology report of biopsy of tumor (and lymph node(s), if applicable) Final

pathology report from **each** performed surgery

OR notes from surgeon (and reconstructive surgeon if applicable) for **each** procedure

Radiology report of specimen mammogram (if applicable)

Initial consult notes from Radiation Oncology and Medical Oncology for clarification purposes can be helpful.

Additional documents needed for patients undergoing neoadjuvant chemo Post

neoadjuvant therapy (preop) surgeon note detailing physical exam Post

neoadjuvant therapy mammogram report (if done)

Post neoadjuvant therapy ultrasound report (if done) Post

neoadjuvant therapy CT report (if done)

Post neoadjuvant therapy MRI report (if done)

General Instructions

For fields where Single Select or Multi-Select apply, the default in the database is "Select an option". This should indicate to the abstractor that the field is incomplete and some action is required to complete the information.

For fields where the instructions call for the entry of a numeric value, please enter
–99 for any value that is missing, not recorded or not applicable.

1 Patient Demographics

****Items in 'red' are HIPAA Non-transferable Data Elements - - Study Site use only****

1.1 ***Field:*** Patient Study ID

Response Options: 5 digit identification number

Instructions: Enter a numeric value

Location: Created by site programmer

Definition: Unique random number assigned by the programmer. The first digit should identify the site (see Hospital/Study Site) and the last 4 digits should identify individual at that site. First digit: 2=UVM 3=GH 4=KPCO 5=Marshfield

1.2 ***Field:*** **Patient Last Name**

Response Options: Text

Instructions: Record text value

Location: Medical Record

Definition: Patient's last name at the time of initial surgery. Only provided to chart abstractor to aid in identification of the patient.

1.3 ***Field:*** **Patient First Name**

Response Options: Text

Instructions: Record text value

Location: Medical Record

Definition: Patient's first name at the time of initial surgery. Only provided to chart abstractor to aid in identification of the patient.

1.4 ***Field:*** **Patient Middle Initial**

Response Options: Text

Instructions: Record text value

Location: Medical Record

Definition: Patient's middle initial at the time of initial surgery. Only provided to chart abstractor to aid in identification of the patient.

1.5 ***Field:*** **Date of Birth**

Response Options: Date

Instructions: Record mm/dd/yyyy

Location: Medical Record

Definition: Used to calculate Patient's age at time of first surgery. Only provided to chart abstractor to aid in identification of the patient.

1.6 ***Field:*** Patient Age at time of 1st surgery

Response Options: Not abstracted field-Calculated by programmer

Instructions: Not abstracted field-Calculated by programmer

Location:

Definition: Age at the time of initial surgery. Calculated by programmer by subtracting the date of birth from the date of initial surgery using the following algorithm:

PatientAge=intck('YEAR', *bdate*, *date of initial surgery*)

If no date of initial surgery is available, we will default to age at diagnosis, based on likelihood of surgery within 30-60 days of diagnosis.

1.7 ***Field:*** **Patient Address**

Response Options: Text

Instructions: Record full address

Location: Medical Record

Definition: Patient's full address closest-in-time-to 30-jun-1999. Only used by programmer for geocoding.

1.8 ***Field:*** **Geocode**

Response Options: Not abstracted - - calculated by programmer from address

Instructions: Not abstracted - - calculated by programmer from address

Location:

Definition: Census data applied to the best possible geocode for the address closest-in-time-to 30-jun-1999. In the VDW methodology the Census2000 file consists of "2000 Census Demographics for the set of people with a geocodable address in the monthly memberships extract files. (For people w/>1 address we used the one they would have been found at on or closest-in-time-to 30-jun-1999.) Only used by programmer to generate summary variables. Sites without VDW census files need to capture an address closest-in-time-to 30-jun-1999 that can be geocoded to census block, block group, tract, county or zip code and obtain permission to get the addresses geocoded and associated with 2000 US Census data.

1.9 ***Field:*** Map flag

Response Options: B=Block

G=Block group

T=Tract

C=County

Z=Zip code

U=Unable to be mapped

Instructions: Character code - - Not abstracted - - calculated from address

Location: To be added by the site programmer

Definition: Level at which the address data were mapped.

1.10 ***Field:*** Median Family Income

Response Options: Integer - - Not abstracted - - obtained from geocode

Instructions: Integer - - Not abstracted - - obtained from geocode

Location: 2000 Census Data

Definition: Derived from the 2000 census data as associated with the geocoded address. Summary of median family income from geocode data (var P077001)

1.11 ***Field:*** Education3

Response Options: xxx.x% - - Not abstracted - - obtained from geocode

Instructions: xxx.x% - - Not abstracted - - obtained from geocode

Location: 2000 Census Data

Definition: % of the population age 25+ that graduated high school. Derived from the 2000 census data as associated with the geocoded address. Sum(P037011, P037028) /sum(P037002, P037019)

1.12 ***Field:*** Education6

Response Options: xxx.x% - - Not abstracted - - obtained from geocode

Instructions: xxx.x% - - Not abstracted - - obtained from geocode

Location: 2000 Census Data

Definition: % of the population age 25+ that graduated college.

Derived from the 2000 census data as associated with the geocoded address. Sum(P037015, P037032)/ sum(P037002, P037019)

1.13 ***Field:*** Patient Height

Response Options: Height in inches “xx.x”; if height is missing code as -99

Instructions: Record the first height closest to the date of the initial surgery. 1 Foot = 12 inches. If recorded in centimeters, convert: cm x 0.39 = inches

Location: Medical Record

Definition: Patient Height in inches

1.14 ***Field:*** Patient Weight

Response Options: Weight in pounds “xxx.x”; If weight is missing, code as -99

Instructions: Record the weight closest to the date of initial surgery but no earlier than 6 months prior. If this is missing, record the weight closest to the date of initial surgery, but no later than 6 months post-surgery. If chart weight recorded in kilograms, convert: kg x 2.2 = lbs.

Location: Medical Record

Definition: Patient weight in pounds

1.15 ***Field:*** Patient BMI

Response Options: "xxx.x" - - Not an abstracted field - - calculated by programmer; If either weight or height are missing, BMI will be missing and should be recorded as -99.

Instructions: "xxx.x" - - Not an abstracted field - - calculated by programmer; If either weight or height are missing, BMI will be missing and should be recorded as -99.

Location:

Definition: Body Mass Index

BMI(kg/m^2)=(weight in pounds * 703)/height in $inches^2$

1.16 ***Field:*** Gender

Response Options: Female

Male

Transgender

Other

Not Recorded

Instructions: Select one value

Location: Medical Record

Definitions: Patient gender

1.17 ***Field:*** Ethnicity

Response Options: Hispanic

Non-Hispanic

Not Recorded

Instructions: Select one value

Location: Medical Record

Definitions: Hispanic is defined as a person of Mexican, Puerto Rican, Cuban, South American (except Brazil) or Central American origin, regardless of race.

1.18	***Field:***	Race
	Response Options:	White<br>African American<br>American Indian/Alaskan Native<br>Asian<br>Native Hawaiian/Pacific Islander<br>Not Recorded
	Instructions:	Select one value
	Location:	Medical Record
	Definitions:	White refers to people having origins in any of the original peoples of Europe, the Middle East or North Africa.<br>African-American refers to any of the black racial groups of Africa including Caribbean-Americans.<br>American Indian or Alaskan Native refer to people having origins in any of the original peoples of North, Central or South America, and who maintain tribal affiliations or community attachment.<br>Asian refers to people having origins in any of the original peoples of the Far East, Southeast Asia or the Indian subcontinent, for example Cambodia, China, India, Japan, Korea, Malaysia, Pakistan, the Philippine Islands, Thailand and Vietnam.<br>Native Hawaiian or Pacific Islander refer to people having origins in any of the original peoples of Hawaii, Guam, Samoa, or other Pacific Islands.<br>If more than once race is recorded use the following guidelines. If race is recorded as white and another race, code as the other race. If a women’s race is recorded as Hawaiian and another race, code as Hawaiian. Otherwise code as the first non-white race listed. This hierarchy is based on the instructions provided in the SEER coding manual (1998).

1.19 ***Field:*** Insurance

Response Options: Commercial

Medicare

Medicaid

Private Pay

Other

Instructions: Select all that apply - - Multi-select

Location: Medical Record

Definition: Commercial coverage = Any employer groups, or federal/state groups. Record coverage closest to the date of initial surgery but no earlier than 6 months prior. If this is missing record coverage closest to the date of initial surgery but no later than 6 months post-surgery.

Medicaid coverage = Code coverage through any state-specific low income plan (e.g., Washington State's Basic Health Plan)

Private Pay coverage = (mostly Individual & Family)

Other coverage = Participant had insurance, but it is unclear what type.

1.20 ***Field:*** Hospital / Study Site

Response Options: Numeric - - Same identifier as the first digit of the patient id

Instructions: Select one value

Location: To be added by site programmer

Definition: 2=UVM; 3=Group Health; 4=KPCO; 5=Marshfield

2 Diagnostic Event

2.1 ***Field:*** Diagnosis Date

Response Options: Date

Instructions: Record mm/dd/yyyy

Location: Pathology Report, Radiology Report, Pre-Operative Consultation or Clinical Exam

Definition: If known malignancy pre-op, this is the date of the first pathology report identifying a new breast cancer or positive lymph node with no breast pathology.

If unknown malignant diagnosis pre-operatively, (highly suspicious for malignancy, but no biopsy) this is the date of the first radiology report suggesting malignancy (BIRAD 5), or clinical exam (whichever is earlier) that describes a suspected malignancy with the need for a cancer operation.

For concerning breast mass only, we will default to date of surgeon's pre-operative consultation.

2.2 ***Field:*** Pre-Operative Diagnosis Type

Response Options:

Known malignancy – DCIS

Known malignancy – IDC

Known malignancy – ILC

Known malignancy – FNA+

Known malignancy – Malignant axillary node of suspected primary

Known malignancy – Type unknown

Known malignancy – Stage IV

Unknown malignant diagnosis

Instructions:

For all selections except "unknown malignant diagnosis" this field should be identified by pathology report. Where differences between clinic records and pathology report exist, highest credence to pathology report for diagnosis type

Select one value

Location: Pathology Report, Radiology Report, Pre-Operative Consultation or Clinical Exam

Definition:

DCIS = Ductal Carcinoma in Situ

IDC = Infiltrating or Invasive Ductal Carcinoma

ILC = Infiltrating or Invasive Lobular Carcinoma

FNA = Fine Needle Aspirate

Known malignancy-FNA + If only an FNA is done (which can only determine the presence of malignant cells, not determine the type), choose this option.

Known malignancy-malignant axillary node of suspected primary-Choose this option ONLY if there is no breast biopsy showing malignancy.

For cases of known pre-operative malignant diagnosis, select the most concerning confirmed pathology identified on pathology report. Always select invasive diagnosis over in-situ disease. For example, when pathology report lists two breast malignancy diagnoses:

For DCIS and IDC—choose IDC

For DCIS with confirmed micro invasion—choose IDC

For DCIS with suspected micro invasion—choose DCIS

For IDC and ILC—choose predominant histology, if neither is noted as predominant, defer to IDC

For Multifocal breast cancer with two different co-existing invasive histologies, code diagnosis type according to the larger of the two lesions.

**2.3**	***Field:***	Indication for Surgery
	Response Options:	Abnormal physical exam
		Concerning pathology on previous biopsy (core, excisional or FNA)
		Abnormal/concerning mammogram
		Abnormal/concerning MRI
		Abnormal/concerning Ultrasound
		Not Applicable
	Instructions:	Select all that apply ** this field is ONLY intended to indentify reasons for surgery when Field 2.2="Unknown malignant diagnosis". For known malignant diagnosis, abstractors can automatically select "Not Applicable" only.
	Location:	Surgical Consultation Note
		Radiology reports of all imaging modalities performed
	Definition:	******If known malignancy of any type is selected for 2.2, select "Not Applicable" for this response as the only response for this question.<br>Otherwise, this is to be filled out if there is no path report prior to surgery demonstrating malignancy. This field helps distinguish the indication for surgery, when selection "unknown diagnosis" is selected for field 2.2.<br>Previous pathology that would be considered "concerning" and commonly utilized as an indication for breast re-excision include atypical ductal hyperplasia, atypical papilloma, atypical lobular hyperplasia, or LCIS

**2.4**	***Field:***	Laterality
	Response Options:	Right
		Left
	Instructions:	Select one value
	Location:	Pathology, Radiology and/or Surgeon initial consult
	Definition:	If patient is diagnosed with bilateral breast cancer, **<u>two</u>** separate forms need to be completed. Each individual breast is treated as a unique entity for risk of close or positive margins and choice of operative procedure. If there is a discrepancy between existing medical records regarding laterality, please work with project manager to identify correct side. Generally if the majority of records 2/3 or 3 of 4 identify left, then choose left.

**2.5**	***Field:***	Neoadjuvant Treatment
	Response Options:	No<br>Yes – chemo<br>Yes – chemo/radiation<br>Yes – endocrine
	Instructions:	Select one value
	Location:	Surgeon, Medical Oncologist and/or Radiation Oncologist consults
	Definition:	No = patient had surgery as first treatment<br>Yes - chemo = patient had chemotherapy before surgery<br>Yes - chemo/radiation = patient had chemotherapy and radiation treatment before surgery<br>Yes - Endocrine = patient had hormonal treatment such as Tamoxifen or Arimidex before surgery**<br>Select the best fit for treatment delivered before surgery. If both chemotherapy and endocrine therapy are given, select chemotherapy.<br>**If Endocrine therapy for > 6 months for reasons of elderly or patient co-morbidities, these patients should be excluded from data base (this represents “salvage surgery” rather than “neoadjuvant” with an initial intent to perform surgery.)

2.6 ***Field:*** Pre-Neoadjuvant Treatment Tumor Size

Response Options: "xxx.x" - - record in millimeters

Instructions: Record numeric value

If field "Neoadjuvant Treatment" = No; enter -99

Location: Surgeon initial consult

Pre-operative radiology reports

Definition: This field is intended to give an estimate of the original tumor size, for those patients who were treated with some form of neoadjuvant therapy. This is meant to record the tumor size at its largest, prior to any ***treatment given.*** Use the surgeon initial consult or any available radiology reports prior to surgery. Record largest reported size from any report prior to treatment in millimeters, for clinical estimation of pre-neoadjuvant treatment tumor size.

If patient did not have any neoadjuvant treatment and went directly to surgery, record "-99"

If no actual tumor size is given, and only described with adjectives, or comparisons to objects, e.g.: "small", "cluster of microcalcification", "walnut sized" or "large", then record -99.

If no size at all mentioned then record -99

If a size range is described "3-4 cm", then record the largest size given in mm.

If two different sizes are recorded, select the larger size reported.

2.7 ***Field:*** Pre-Neoadjuvant Therapy Imaging

Response Options: N/A – No Neoadjuvant Treatment

Mammography

CT Scan

Ultrasound

MRI

Instructions: Select all that apply - - Multi-select field

Location: Radiology reports before the initiation of chemotherapy

Definition: This field is meant to define any imaging modality used to obtain the pre neoadjuvant therapy tumor size.

2.8 ***Field:*** Suspected Inflammatory Breast Cancer

Response Options: No

Yes – biopsy proven dermal extension

Yes – no biopsy proven dermal extension

Instructions: Select one value

Location: Surgeon initial consult note, Radiation consult note, or medical oncologist consult note. Do not use radiology report. Pathology report of skin biopsy, if applicable.

Definition: No=No mention in the clinical exam that this is suspicious for inflammatory cancer.

Yes-biopsy proven dermal extension= Suspected inflammatory breast cancer by initial consult notes and a skin punch was performed which showed tumor in the skin or dermal lymphatics.

Yes—no biopsy proven dermal extension=Choose this option if there is documentation of clinical suspicion of inflammatory cancer, however no biopsy was performed which showed tumor in the skin or dermal lymphatics, or a biopsy was performed but did not show tumor in the skin or dermal lymphatics.

Special Instructions:

Use the initial consult note of surgeon, and if needed, radiation and medical oncologists consult notes to determine if one or more physicians identify this as inflammatory breast cancer in their clinical exam. Do not use radiology reports as identifiers for inflammatory breast cancer. Key words to watch for in the clinical exam are skin redness, skin edema, peau d'orange, and/or skin thickening leading to "clinically suspicious for inflammatory cancer." Occasionally skin edema is described as "peau d'orange". Typically 95% of inflammatory breast cancer will receive neoadjuvant chemotherapy. If you selected "Yes" for inflammatory and "No" for neoadjuvant therapy, case should be reviewed.

2.9 ***Field:*** Multifocal Breast Tumors

Response Options: No

Yes—same quadrant, biopsy proven preop

Yes—same quadrant, no biopsy proven preop

Yes—multiple (multicentric) quadrants, biopsy proven preop

Yes—multiple (multicentric) quadrants, not biopsy proven preop

Yes - unknown same vs. multiple (multicentric) quadrants

Instructions: Select one value

Location: Surgeon's pre-operative consultation; pre-operative radiology reports combined with pre-operative radiology reports (based on core biopsies only)

Definition: **Multifocal** and **multicentric** can be confusing terms. If two or more distinct areas of concern are identified in the same quadrant, this is **"multifocal"**. The term **"multicentric"** is generally reserved to describe two or more foci of breast cancer in different quadrants of the same breast.

Multifocal breast tumors refer to the "pre-operative" suspected condition. This may contribute to surgeon/patient decision for mastectomy. If using pathology report only initial biopsies prior to definitive surgery should be used.

Yes—same quadrant, biopsy proven preop. Choose this if the patient has two or more biopsy proven cancers that are in the same quadrant.

Yes—same quadrant, not biopsy proven preop. Choose this if the patient has two or more tumors which are highly suspicious, but <u>not</u> proven by biopsy, and are in the same quadrant.

Yes—multiple (multicentric) quadrants, biopsy proven preop. Choose this if the patient has two or more biopsy proven cancers that are in different quadrants.

Yes—multiple (multicentric) quadrants, not biopsy proven preop. Choose this if the patient has two or more tumors which are highly suspicious but <u>not</u> proven by biopsy, and are in different quadrants.

Yes – unknown same vs. multiple (multicentric) quadrants. Choose this if records indicate "multifocal", but no mention anywhere of same vs. multiple quadrants.

2.10 ***Field:*** Multifocal Breast Tumors Identified By

Response Options: Mammogram

MRI

Ultrasound

Not Applicable

Instructions: Select all that apply - - multi-select field

Location: Radiology reports of all imaging modalities performed

Definition: These are commonly performed imaging studies for newly diagnosed breast masses or cancer.

CT= computed tomography, also known as CAT scan, and is generally only done for staging of breast cancer when there is a high incidence of suspicion of metastatic disease. Sometimes, a breast mass is first identified on CT scan when the CT scan was originally performed for some other indication (i.e. further characterization of a lung nodule seen on Chest x-ray)

MRI = Magnetic Resonance Imaging

Not Applicable: select Not Applicable if patient does not have multifocal breast tumors, field 2.9=”No”

2.11 ***Field:*** Previous Breast Cancer

Response Options:

No

Yes - Contralateral breast, before 01/01/03

Yes – Contralateral breast, after 01/01/03 and surgery performed at this institution

Yes – Contralateral breast, after 01/01/03 and surgery performed elsewhere

Yes – Contralateral breast, after 01/01/03 and no surgery performed

Yes - Ipsilateral breast, before 01/01/03

Yes – Ipsilateral breast, after 01/01/03 and surgery performed at this institution

Yes – Ipsilateral breast, after 01/01/03 and surgery performed elsewhere

Yes – Ipsilateral breast, after 01/01/03 and no surgery performed

Yes- Bilateral cancer, regardless of date

Instructions:

Select one value

If patient has had both a previous right-sided breast cancer and a left-sided breast cancer, select YES- bilateral cancer. These cancers may have occurred at same or different time periods.

Location: Initial consult note of surgeon. May need radiology reports to determine location of prior cancer

Definition:

All prior breast cancers, whether contralateral or ipsilateral should be entered into the database

No = Patient has not had any prior breast cancer

Contralateral= opposite breast

Ipsilateral= same breast

Bilateral=both prior right and left breast cancers

2.12 ***Field:*** Previous Chest / Breast Radiation

Response Options: No

Yes – Breast Cancer

Yes – Other Cancer/Other Reason

Instructions: Select one value

Location: Surgeon’s initial consult or Radiation Oncology consult

Definition: No—Choose this if there is no documentation that the patient has ever received chest/breast radiation.

Yes—Breast Cancer—Choose this if there is indication that the patient received prior radiation therapy for breast cancer.

Yes—Other cancer/other reason—Chose this if the patient received previous radiation to the chest or axilla for a reason other than breast cancer,(i.e. mantle radiation for Hodgkin’s Lymphoma.) This should be found in the surgeon’s initial consult, or a Radiation Oncology consult.

2.13 ***Field:*** Node Status Pre-Treatment

Response Options: Negative without pathologic confirmation Negative with pathologic confirmation by FNA Negative with pathologic confirmation by SLN Negative with unknown pathologic confirmation Positive without pathologic confirmation

Positive with pathologic confirmation by FNA

Positive with pathologic confirmation by SLN

Positive with unknown pathologic confirmation

Instructions: Select one value

Location: Surgeon's initial consult or Radiation Oncology consult

Medical oncology consult, initial pathology biopsy report(s)

Definitions: This field refers to the clinical suspicion of node status prior to initiating any definitive cancer therapy (cancer-directed surgery or chemo/radiation). This field is not for recording the results of Nodal Surgery (see Section 7). The single exception would be if SLN biopsy is done before neoadjuvant therapy.

The pathology report documenting a positive node may be in the original pathology report from a breast biopsy if radiology also performed a biopsy of the node at the time of initial diagnosis. For patients who undergo neoadjuvant therapy, some centers perform the SLN biopsy before initiating chemotherapy. Other times, a biopsy of the node may be performed in the office by the surgeon.

If the patient has had a previous axillary dissection and there is no clinical suspicion currently of positive nodes, choose "negative without pathologic confirmation."

FNA = Fine needle aspiration

SLN = Sentinel lymph node

Negative without pathologic confirmation—Choose this if the patient is clinically node negative by surgeon's physical exam. May be recorded in the clinical notes as "clinical N0."

Negative with pathologic confirmation by FNA—Choose this if an FNA is done of a lymph node and the results are negative for malignancy.

Negative with pathologic confirmation by SLN—Choose this if a sentinel lymph node biopsy is performed before breast surgery or neoadjuvant chemotherapy, and the results are negative for malignancy.

Positive without pathologic confirmation—Choose this if the surgeon and/or other oncologists' notes, or any imaging modality reports state that lymph nodes are clinically positive, but there is no biopsy performed to prove or disprove this. If

clinician indicates nodes are “suspicious”, please categorize as “Positive without pathologic confirmation”.

Positive with pathologic confirmation by FNA—Choose this if an FNA is done of a lymph node and the results are positive for malignancy.

Positive with pathologic confirmation by SLN—Choose this if a sentinel lymph node biopsy is performed before breast surgery or neoadjuvant chemotherapy, and the results are positive for malignancy.

2.14 ***Field:*** Pre-Operative Estimated Breast Tumor Size – Physical Exam

Response Options: Numeric “xxx.x”

Instructions: Record in millimeters “xxx.x”; If tumor size is not recorded, enter -99

Location: Only Surgeon initial consult if patient did not receive neoadjuvant therapy.

If patient did receive neoadjuvant therapy, also need the last preoperative clinical note that contains physical exam with physical exam estimated tumor size.

Definition: This is the measurement of the tumor size by last clinical exam noted before breast operation.

If neoadjuvant therapy=No, then this will be the only place a physical exam estimated size of tumor is recorded.

If neoadjuvant therapy=Yes, this value is meant to record the response to neoadjuvant therapy, and should represent the last recorded physical exam size prior to surgery.

If no actual tumor size is given, and only described with adjectives, or comparisons to objects, e.g.: “small”, or “walnut sized” or “large”, then record -99.

If no size at all mentioned the record -99

If a size range is described “3-4 cm”, then record the largest size given in mm.

If size is recorded as “< 1 cm” record as 9mm.

If size is recorded as “< 5 mm” record as 4 mm.

2.15	***Field:***	Pre-Operative Imaging
	Response Options:	Mammography<br>CT<br>Ultrasound<br>MRI
	Instructions:	Select all that apply - - Multi-select field
	Location:	Radiology reports of all imaging modalities performed
	Definition:	These are commonly performed imaging studies for newly diagnosed breast masses or cancer.<br>CT= computed tomography, also known as CAT scan, and is generally only done for staging of breast cancer when there is a high incidence of suspicion of metastatic disease. Sometimes, a breast mass is first identified on CT scan when the CT scan was originally performed for some other indication (i.e. further characterization of a lung nodule seen on Chest x-ray)<br>MRI = Magnetic Resonance Imaging

2.16 ***Field:*** Pre-Operative Estimated Breast Tumor Size - Imaging

Response Options Numeric “xxx.x”

Instructions: Record in millimeters “xxx.x”;

If tumor size is not recorded, enter -99

If neoadjuvant therapy=YES, and no additional imaging performed, enter -99

Location: All radiology reports and breast imaging prior to surgery.

Definition: Record the largest tumor size as best documented in radiology studies performed before operation. If there exist discrepancies between reports, record the largest reported tumor size. If the patient has more than one tumor identified, use the measurement of the largest tumor. If the report says “2 to 3 cm”, enter the largest measurement, in this case it would be “30 mm.” If there are multiple reports with measurements for both pre and post biopsy, defer to the measurement of the tumor pre-biopsy, as there can be distortion and hematoma surrounding the tumor post-biopsy, making measurements less accurate. If the only measurement obtained is post-biopsy, enter that measurement.

Mammography—Consult both screening/diagnostic and additional view reports, if measurements are listed on both, defer to the measurement on the additional view report. If the report identifies a measured region or distance of concern for malignancy, rather than a measured mass, record that distance in millimeters. For example, if the report says “suspicious calcifications over a 6 centimeter area”, enter 60 mm as reported tumor size.

CT scan—If a patient is having a staging chest CT, there may be mention and measurement of a tumor in the breast.

Ultrasound - If there are ultrasound measurements from both a surgeon and the radiologist, defer to the radiology report.

MRI = Magnetic Resonance Imaging. Consult radiology report.

Use caution in reading these reports. There may be mention and measurements of many findings such as cysts and benign solid tumors. Be sure the measurements entered are of the malignancy or suspected malignancy.

Special instructions for patients undergoing neoadjuvant therapy:

This section is meant to record the response to therapy in these patients, and the best estimated tumor size at the conclusion of neoadjuvant treatment, which is typically the tumor size on which surgical treatment is based. Do not simply re-enter the tumor sized recorded in the data field “pre-neoadjuvant therapy tumor size-imaging”.

If repeated imaging was done but only describes the tumor as “unchanged, or “smaller”, then enter -99.

2.17 ***Field:*** Final Treatment Diagnosis

Response Options: DCIS

IDC

ILC

Instructions: Select one value

Location: Pathology Reports, Tumor Registry, and post-operative or surveillance surgical or oncology clinic notes.

Definition: This is the tumor type for which the patient was ultimately treated for this diagnostic event. In almost all cases in which there is some element of invasive tumor (IDC or ILC) and DCIS, almost always the invasive component, present on any of the pathology reports, is the “Final Treatment Diagnosis.” This is most likely to be the tumor type selected by your hospital’s tumor registrar, and also the tumor type which ultimately dictate the patient’s subsequent treatment course for this diagnostic event.

DCIS = Ductal Carcinoma in situ

IDC = Infiltrating Ductal Cancer

ILC = Lobular Cancer

3 Breast Surgery

Each distinct surgery done for a single diagnosis date for each individual breast requires this section to be completed for each surgery date. For instance, if a patient first underwent a partial mastectomy, followed by a total mastectomy to achieve clear margins, this section would be completed x 2. If there were two partial mastectomies, followed by a total mastectomy, there would be three separate surgery data forms completed.

3.1 ***Field:*** Surgical Procedure Date

Response Options: Date

Instructions: Record mm/dd/yyyy

Location: Operative report

Definition: Month/day/year on which the first surgical procedure was performed on the affected breast. This would be the date of the first surgical excision performed at your hospital/by your surgeon for which the pathology report identified cancer or a previous non-surgical biopsy identified cancer. In most instances where the diagnosis is made by non-surgical biopsy, the procedure date will be the date on which the first cancer-directed surgery was performed.

3.2 ***Field:*** Surgeon ID

Response Options: List and crosswalk available at your site.

Instructions: Select one value

Location: Should be pre-populated; otherwise surgeon's report

Definition: Unique random number assigned by the programmer who will keep a crosswalk to the internal physician identifier that will not be shared beyond the individual site. This should be the surgeon who conducted the initial breast cancer surgery.

3.3 ***Field:*** Facility

Response Options: List and crosswalk available at your site.

Instructions: Select one value

Location: Medical Record

Definition: Name of the facility/hospital/surgical clinic where the initial surgery was done.

3.4 ***Field:*** Breast Procedure Type

Response Options: Breast biopsy (unknown malignant diagnosis pre-op)

Partial mastectomy

Total mastectomy

None

Instructions: Select one value

Location: Operative Report; Surgeon's Pre-operative note

Definition: Breast Biopsy - Breast biopsy should only be selected when the initial surgical procedure was performed without a confirmed pathologic diagnosis of breast cancer pre-operatively. Following are examples of Diagnostic BREAST BIOPSY: Excisional Breast biopsy, non-guided; Excisional Breast biopsy, image guided

Partial Mastectomy - This procedure should only be selected when confirmed malignancy pre-operatively by pathology, or surgeon pre-operative or operative note indicates that he/she is treating this as a malignant diagnosis.
Following are examples which would be coded as PARTIAL MASTECTOMY:

- Lumpectomy
- Partial mastectomy without axillary lymph node dissection
- Partial mastectomy with sentinel node biopsy
- Partial mastectomy with axillary lymph node dissection
- Partial mastectomy with nipple resection
- Segmental mastectomy
- Re-excision of the biopsy site for gross or microscopic residual disease
- Any of above with image guidance

Total Mastectomy - This procedure should only be selected when confirmed malignancy pre-operatively by pathology, or surgeon pre-operative or operative note indicates that he/she is treating this as a malignant diagnosis.

Following are examples which would be coded as TOTAL MASTECTOMY

- Subcutaneous mastectomy with or without axillary lymph node dissection
- Modified radical mastectomy
- Simple mastectomy
- Radical mastectomy with partial or full resection of pectoralis major with axillary lymph node dissection
- Mastectomy NOS

NONE: select none when no procedure was ever performed on the breast, but the primary diagnosis of breast cancer

was identified from an axillary procedure and no subsequent surgery was done on the breast ipsilateral to the side of the axillary procedure. This is a relatively unusual circumstance of a breast cancer being identified by a nodal metastasis (approximately 1% breast cancer diagnoses), and no primary tumor ever identified and no surgery done on the affected side (<1%)

3.5 ***Field:*** Anesthesia

Response Options: Local

Monitored Anesthesia Care (MAC)

General

Regional

Not Recorded

Instructions: Select one value. Please select the highest level of anesthesia administered to the patient for conducting the procedure.

Location: Operative Report

Definition: Please select the highest level of anesthesia administered to the patient for conducting the procedure.
Comments:

- Utilization of local anesthetics is common even when general anesthesia is utilized, for improved patient analgesia post-operatively. In cases where local anesthesia is administered in addition to general anesthesia, select: General.
- For patient for whom case was initiated as a local procedure and subsequently converted to a general anesthetic, select: General.

Local = Patients receive local anesthesia and remain awake or sedated for the procedure. Most often the operative note will describe administration of a local anesthetic such as lidocaine, bupivicaine or other local anesthetic agent, with the patient awake. This is typically injected directly into the skin and the portion of affected breast which is to be removed. Local anesthesia is often performed as an office based procedure, routinely given by the surgeon only, and performed in the absence of an anesthetist. Typically local anesthesia use is limited to procedures such as breast excisional biopsy and partial mastectomy, and NOT mastectomy.

For IV sedation, please select Monitored Anesthesia Care (MAC) = This anesthesia typically requires the presence of an anesthetist in addition to surgeon. It involves the administration of sedating or amnestic drugs in addition to local anesthesia, and always requires an IV, continuous oxygen monitoring, and typically requires time in the recovery room under monitored nursing care following the procedure.
Regional = Patients receiving an axillary block, or other type of injected anesthetic regional block that would allow for the procedure to be performed with the patient awake. Generally this is administered by an anesthetist. This has

relatively infrequent utilization, but may be used for patients with higher operative risk from a cardiovascular standpoint.

General = Patient's procedure was performed under conditions of a general anesthetic. This is routinely done under the care of an anesthetist and may be described in the operative report as "general endotracheal" or ""LMA for laryngeal mask airway. There is generally a separate anesthetic record as a component of the patient's procedure. This is the most common anesthetic utilized for procedures involving full removal of breast (mastectomy) and/or full axillary nodal dissection.

Not Recorded = Select if there is no mention of the type of anesthesia used for the procedure in the operative report. Note: The anesthesia used can often be deduced by reading the operative report.

3.6 ***Field:*** Partial Mastectomy / Breast Biopsy Tumor Localization Technique

Response Options:
Palpation
Mammographic Needle Localization
Ultrasound Needle Localization
MRI Needle Localization
Ultrasound Localization
Gamma Probe Localization
Not Recorded

Instructions: Select one value

Location: Operative Report

Definition: If patient underwent a mastectomy (selected above in Breast Procedure type) select Not Recorded. If patient underwent an initial breast biopsy or a partial mastectomy, complete this section.
Source Documentation: Operative Report, Radiology Reports same day as procedure

Brief background regarding tumor localization:
Surgical goals of breast cancer partial mastectomy are to remove the breast tumor in its entirety with a rim or surrounding normal breast tissue. In order to achieve this, surgeons may use one, two, or several techniques in the operating room to identify the tumor and seek to identify the full extent of the tumor in order to achieve clear margins. Often for smaller tumors (<1.5 cm or so), and almost always for non-palpable tumors identified radiographically, surgeons will utilize some intra-operative method or radiologic method to identify smaller or clinically occult tumors. In some instances surgeons may use several techniques available to them to achieve complete removal of smaller or clinically non-palpable tumors.

Palpation = The surgeon identifies the tumor in the breast by palpation (feels it with their fingers). This is the most common technique. Most often requires that tumors be at least 1 cm in order to palpate, often larger in a larger breasted patient. If no specific technique is described in the operative report and the tumor is greater or equal to 2.0 cm, this can be selected as the default selection.

Mammographic **Needle** Localization = This typically requires placement of wires or flexible needles by the radiology team, or the surgeon at some institutions, and is performed prior to performing the partial mastectomy. The patient typically comes to the operating room with wires already placed in the breast. This is a common technique in the United States for performing partial mastectomy when only microcalcifications on mammogram are the presenting abnormality requiring breast surgery. Typically a radiology report is generated for this procedure.

Ultrasound Needle Localization = This typically requires placement of wires or flexible needles by the radiology team, or the surgeon at some institutions, and is performed prior to performing the partial mastectomy. Generally reserved for small non-palpable lesions OR when all calcification were removed at stereotactic breast biopsy AND a clip was left behind at the biopsy site and can be identified by ultrasonagrapher. The patient typically comes to the operating room with wires already placed in the breast. Typically a radiology report is generated for this procedure.

MRI Needle Localization = This is performed only in select centers currently. This is generally utilized when abnormalities of concern are identifiable on MRI only.

Ultrasound Localization = This is generally performed by the surgeon in the operating room and no wires are placed. The ultrasonagrapher identifies either the existing tumor or possibly a hematoma (blood clot) or previously placed radiologic clip which was placed at the time of surgical
biopsy to better identify the site of the tumor. Ultrasound identification of the tumor or tumor site is almost always described in the operative report.

Gamma probe localization = This is a newer technique where a radioactive tracer is injected at the actual site of the tumor, often prior to surgery. In this procedure, typically an occult primary tumor is identified by utilizing the gamma probe, and complete excision would be described in the operative report by a significant diminishment in radioactivity AT THE SITE OF PRIMARY TUMOR. This is different from a sentinel node procedure where an injection may be done at site of primary tumor to identify the nodal drainage pattern of the primary tumor.

Not recorded = If no specific technique is described in the operative report and the tumor is less than 2.0 cm, this can be selected as the default selection.

3.7 ***Field:*** Surgeon's Intra-operative Margin Assessment

Response Options:

Specimen Mammogram

Specimen Ultrasound

Radiology – NOS

Pathologic Assessment

Specimen Palpation

Not Recorded

Instructions: Select all that apply - More than one technique may be utilized, so this is a multi-select field.

Location: Operative Report, Pathology Report (section on intra-operative assessment), Radiology Report (would only apply to specimen mammogram or specimen ultrasound)

Definition: *Brief background regarding Intra-operative margin assessment:*

This section is to identify any or all techniques (multi-select) that the surgeon uses in the operating room to identify that the tumor has been removed in its entirety. This information is most commonly found in the surgeon's operative report but may also be described in the final pathology report that an intra-operative assessment of margins was performed if one of the techniques utilizing assistance from a pathologist is utilized.

"Not Recorded" should only be selected if there is no description of any technique of inspection of the partial mastectomy specimen after excision or if the patient underwent a mastectomy; see "Breast Procedure Type"

Specimen Mammogram = For patients who underwent a specimen mammogram. This is generally described in the operative report that a specimen mammogram was performed. This would most often be performed in cases of small or clinically occult tumors where the surgeon is attempting to extract the tumor or site of the tumor when a needle localization procedure was performed or a previous clip placement marked the site of the tumor.

Specimen Ultrasound = Ultrasound is utilized in the operating room to assess margins of the tumor. This is not always performed in cases where ultrasound was used to identify the tumor in the operating room, and would need to be specifically identified in the operative report as an evaluation of the specimen with ultrasound. This is not a billable procedure and would not be identifiable by billing codes.

Radiology – NOS (Not Otherwise Specified) = Surgeon comments specimen sent to radiology, but specific radiology

modality cannot be determined.

Pathologic Assessment = This involves the surgeon sending the tumor to pathology for an inspection of tumor margins, and awaiting results of that inspection while the patient remains in the operating room. The pathologist may use a variety of techniques such as “frozen section”, “gross inspection”, cytology imprint or others. This can be described in the surgeon’s operative report and/or the final pathology report as an intra-operative assessment. This is generally described in the operative report as sending the specimen to pathology, and awaiting a verbal confirmation from the pathologist that margins appear to be adequate.

Specimen Palpation = Surgeon feels the tumor to assess that margins of excision appear adequate. This is generally reserved only for palpable tumors. This may not always be performed after excision, and should only be selected if the surgeon specifically describes inspecting the specimen by palpation after excision.

3.8 ***Field:*** Surgeon’s Specimen Orientation

Response Options: Suture/Clip Orientation

Specimen Inking

Routine Breast Cavity re-excision

Not Recorded

Instructions: Select all that apply - - If patient underwent a mastectomy (selected above in Breast Procedure type) select “Not Recorded”

Location: Operative Report, Pathology Report (section on gross specimen description)

Definition: *Brief background regarding Intra-operative margin assessment:*
Typically the surgeon will mark the specimen in some way so as to orient it for the pathologist. Without orientation, it can be difficult for the pathologist to identify which specific margin may be closest for tumor.

Suture/Clip orientation = The surgeon will generally describe orienting the specimen with either suture of various lengths or colors, and will generally describe the associated position of the suture markers on the specimen (e.g. superior, lateral, inferior OR 12 o’clock, 3 o’clock). Similar technique can be used with metallic clips (e.g. 1 clip superior, two clips lateral) If either or both techniques are used, select this option.

Specimen inking = The surgeon inks the specimen themselves with different colored inks in the operating room, with a distinct color identifying a distinct direction of the specimen (e.g. yellow:superior; blue: inferior; black:posterior)

Routine Breast Cavity re-excision: Some surgeons routinely re-excise (without pathology interpretation) the partial mastectomy cavity as a means of attaining a wider margin.

Not Recorded = No description of the surgeon’s oriented specimen for the pathology analysis.

3.9 ***Field:*** Intra-Operative Positive or Concerning Margin(s)

Response Options:

No

Yes – Additional tissue taken

Yes – No additional tissue taken

Yes – Not recorded if additional tissue taken

No intra-operative assessment of margins

Not recorded

Instructions:

Select one value

If patient underwent a mastectomy (selected above in Breast Procedure type) select “Not Recorded”

If patient underwent an initial breast biopsy or a partial mastectomy, complete as indicated.

Location:

Operative Report (usually will describe additional tissue taken, sometime requiring re-opening of incision), Pathology Report (section on intra-operative assessment). This may specifically describe a phone conversation with surgeon communicating close or positive margins. Radiology Specimen Mammogram report, as there may be an indication of “microcalcifications close to edge of specimen” or “clip is at posterior edge” or “all microcalcifications (cals) removed and in center of specimen.

Definition:

Brief background regarding Intra-operative positive or concerning margins:
If the pathologist analyzes the surgical specimen before the patient leaves the operating room, they may identify close (tumor close to cut edge of excised specimen) or positive (tumor noted at cut edge of excised specimen). Typically, the pathologist will notify the surgeon in the operating room and the surgeon may decide to excise more tissue.

No = There was no mention in operative report of positive or concerning margins

Yes – Additional tissue taken = Surgeon specifically dictates that assessment of margins demonstrates as close or positive margin and additional breast tissue excised to try and improve or achieve appropriate clearance at that margin

Yes – No additional tissue taken = Surgeon aware of close or concerning margins but elects to take no additional breast tissue

3.10 ***Field:*** Mastectomy Factors

Response Options: Unilateral

Bilateral – Contralateral Prophylactic Mastectomy

Bilateral – Synchronous Bilateral Cancers

` Not Recorded/Not Applicable

Instructions: Select one value

This field is intended to clarify, for patients ultimately receiving a mastectomy, whether they underwent unilateral or bilateral mastectomy and indication. For final procedure=partial mastectomy, select "Not Applicable".

Location: Operative Report

Definition: Mastectomy may be described with the following terms: "Total mastectomy", "simple mastectomy", "modified radical mastectomy" or "skin sparing mastectomy".

Unilateral = Mastectomy performed only on the affected breast for treatment of breast cancer. This can be an 'initial' mastectomy, as the first procedure or a 'subsequent' mastectomy if following initial partial mastectomy where margins were positive or close.

Bilateral – Contralateral Prophylactic Mastectomy. Also described as "mastectomy with removal of uninvolved contralateral breast". The contralateral breast does not have any confirmed malignant diagnosis, but patient elects to remove the opposite breast by personal selection.

Bilateral – Synchronous Bilateral Cancers = If there is a contralateral breast cancer select this option. The patient should have a completed data collection sheet for the opposite breast.

Not Recorded/Not Applicable = Patient has not undergone initial or subsequent mastectomy. This would be the selection of choice for patients undergoing partial mastectomy as the final procedure.

3.11 ***Field:*** Breast Reconstruction

Response Options:

Tissue

Implant

Combined Tissue & Implant / NOS

Not Performed

Not Recorded/Not applicable

Instructions:

Select one value - - Complete for patients undergoing initial or subsequent mastectomy for this instance of cancer diagnosis.

This field is intended to better delineate, for patients undergoing a mastectomy, whether or not there is documentation of breast reconstruction, and if so, what type.

Location: Surgeon's report

Definition:

Not Performed = Medical records indicate patient had a mastectomy and that no reconstruction was ever performed. Subsequent physical exams may indicate "surgically absent breast".

Not Recorded/Not Applicable = It is unclear for patient undergoing mastectomy as to whether patient ever had breast reconstruction. No mention in available documents. If final procedure is a **partial** mastectomy, select "Not Recorded/Not Applicable"

Tissue – Muscle Flap TRAM = Almost always performed by a plastic surgeon. A Trans Rectus Abdominus Muscle (TRAM) is performed, rotating abdominal muscle into the subcutaneous space created by breast removal. This would be categorized as "reconstruction, tissue"

Tissue– Muscle Flap Latissimus Dorsi = Done at same time as mastectomy, or immediately following under same anesthetic. Latissimus Dorsi muscle is rotated on a vascular pedicle from the patient's back to re-create the breast. This would be categorized as "reconstruction, tissue"

Implant - One Stage implant reconstruction. This typically involves implantation of a saline and previously silicone implant that is chosen to best match the size of the opposite breast. This would be categorized under "reconstruction, implant".

Implant - Tissue Expander = Done at the same setting as the mastectomy, most often two surgeons involved. Generally the plastic surgeon implants a tissue expander, often below the pectoralis muscle, which will allow for saline to gradually be injected and allow sequential expansion of tissues.

Combined Tissue & Implant / NOS – Combination of implant (as above) with a tissue transfer procedure (as above). NOS (Not otherwise specified) – Electronic records do not indicate which type of reconstruction.

4 Margins

4.1 ***Field:*** Direction Closest uninvolved margin- invasive carcinoma

Response Options:

Superior

Inferior

Anterior

Posterior

Medial

Lateral

≥10 mm

Cannot be assessed

Not applicable

Instructions:

Select one value

The selection should be chosen which best represents the most concerning margin for which a surgeon may choose to re-operate. Occasionally several margins will be listed as equally close. In that case, select any of the listed radial margins (medial, lateral, superior, inferior).

If patient underwent a mastectomy and margins are not listed (a common practice) select “cannot be assessed”.

Location: Pathology Report

Definition:

Uninvolved margins indicate that there is no tumor seen at the inked pathologic margin. For uninvolved margins, record the direction of the closest margin as recorded on pathology report; only record direction if closest distance if less than 10 mm, if greater than or equal to10 mm do not record direction (i.e. superior, inferior, etc), select “≥10 mm”

- If two margins are equally close, select any radial margin (medial, lateral, superior, inferior). If a radial margin is identified as one of the equally close margins.
- If the two equally close margins are anterior and posterior, select anterior.
- If the two margins that are equally close involve a radial margin and either anterior or posterior, select the radial margin.

- If more than two margins are listed as equally close, then select any of the listed radial margins

If there is not a comment on the pathology report regarding the direction of the closest margin, but a measurement of the closest margin is given, select “cannot be assessed” for direction.
If no comment on margins whatsoever, select “cannot be assessed”.
If final diagnosis =DCIS, choose “Not applicable”
Anterior – Generally refers to toward the skin
Posterior – generally refers to toward the chest wall or pectoralis muscle. “Deep” margins should be considered the same as posterior.
Lateral – refers to toward the axilla Medial – refers to toward the sternum Superior – refers to toward the clavicle
Inferior – refers to toward the abdomen

4.2 ***Field:*** Distance from closest margin – Uninvolved by invasive carcinoma

Response Options: Numeric “xxx.x”

Instructions: Record distance in millimeters “xxx.x”; If there is no comment on margins whatsoever, enter –99. If field 4.1 Direction Closest uninvolved margin- invasive carcinoma, was ≥10mm, enter -99. If field 4.1 was “cannot be assessed”, enter -99. If field 4.1 was “not applicable”, enter -99.

If pathologist uses <1mm, please input 0.9mm. If uses <2mm, input 1.9mm, etc.

Location: Pathology Report

Definition: Uninvolved margins indicate that there is no tumor seen at the inked pathologic margin. For uninvolved margins, record the distance of the closest margin as recorded on pathology report.
If distance is not given, or is listed in another fashion than distance from margin, list (-99).
If patient had mastectomy following a previous partial mastectomy and there is no margin distance listed, do not interpret margin distance if not on path report, and select (-99).

4.3 ***Field:*** Direction closest margin - Uninvolved by DCIS (if present)

Response Options:
Superior
Inferior
Anterior
Posterior
Medial
Lateral
≥10mm
Cannot be assessed
Not applicable

Instructions: Select one value

Definition: Uninvolved margins indicate that there is no tumor seen at the inked pathologic margin. For uninvolved margins, record direction of the closest margin as recorded on pathology report; only record direction if closest distance if less than 10 mm, if greater than or equal to 10 mm do not record direction (i.e. superior, inferior, etc), select "≥10 mm"

This field applies for DCIS margins. DCIS margins should always be commented on when DCIS is the final diagnosis, but DCIS may also be a pathologic component of invasive cancers. Therefore, for invasive cancers, there may be a margin assessment for both the invasive component as well as the DCIS component.

If there is not a comment on the pathology report regarding the **direction** of the closest DCIS margin, but a measurement of the closest DCIS margin is given, select "cannot be assessed" for direction.

If no comment on DCIS margins whatsoever, select "cannot be assessed".

If no mention of DCIS whatsoever on pathology report (invasive tumor only), select "not applicable".

4.4 ***Field:*** Distance from closest margin - Uninvolved by DCIS

Response Options: Numeric "xxx.x"

Instructions: Record distance in millimeters "xxx.x"; if no comment on DCIS margins whatsoever, enter -99. If field 4.3 Direction Closest uninvolved margin- invasive carcinoma, was ≥10mm, enter -99. If field 4.3 was "cannot be assessed", enter -99. If field 4.1 was "not applicable", enter -99.

If pathologist uses <1mm, please input 0.9mm. If uses <2mm, input 1.9mm, etc.

Location: Pathology Report

Definition: For uninvolved margins, record distance of closest margin as recorded on pathology report

4.5 ***Field:*** Direction Margin(s)- involved by invasive carcinoma

Response Options:

Superior

Inferior

Anterior

Posterior

Medial

Lateral

Multiple

Cannot be assessed

Not Applicable

Instructions: Select one value

Location: Pathology Report

Definition: Margins are recorded as involved if so stated on the path report, or if listed as a "positive" margin, or "tumor at inked margin".

For involved margin(s), please record the direction of the involved margin (i.e.: superior, inferior, anterior, posterior, medial, lateral etc.) If more than a single margin is involved, please select: "multiple".

If final diagnosis is DCIS only, select "not applicable"
If there is not a comment regarding the **direction** of the involved margin, but involved or "positive" margin is stated, select "cannot be assessed" for direction.

4.6 ***Field:*** Direction Margin(s) - involved by DCIS

Response Options:

Superior

Inferior

Anterior

Posterior

Medial

Lateral

Multiple

Cannot be assessed

Not Applicable

Instructions: Select one value

Location: Pathology Report

Definition: Margins are recorded as involved if so stated on the path report, or if listed as "positive" margin, or "tumor at inked margin".
For margins involved, please record the direction of the involved margin (i.e.: superior, inferior, anterior, posterior, medial, lateral etc.)
If more than a single margin is involved, please select: "multiple".
This field applies for DCIS margins. DCIS margins should be commented on when DCIS is the final diagnosis, but may also be a component of invasive cancers. Therefore, for invasive cancers, there may be a margin assessment for both DCIS as well and should be recorded in this field
If there is not a comment on the pathology report regarding the **direction** of the positive DCIS margin, but a positive DCIS margin is stated, select "cannot be assessed" for direction.

If no comment on DCIS margins whatsoever, select "cannot be assessed".

If margins not involved by DCIS, select "Not Applicable"

5 Tumor Information

The largest contiguous focus of tumor identified on pathology report is considered the primary tumor. If there is invasive breast cancer and in-situ breast cancer (DCIS) present, the "Maximal Pathological Tumor Size" should be recorded as the invasive tumor size. The in-situ component may also have a measured size as well, which we will record under field "Maximal Extent of Disease". If there is no invasive component
and only in-situ carcinoma (DCIS) reported, then the "Maximal Pathological Tumor Size"
field should be used to describe the size of DCIS.

5.1 ***Field:*** Residual Tumor in Surgical Specimen

Response Options: No

Yes

Instructions: Select one value

Location: Pathology report

Definition: This field is to help identify surgeries performed for pre-operatively biopsy proven cancers where the pathology reports no additional cancer is found in the surgically excised breast tissue. This can occur following a core biopsy (mammatome, other) in which cancer is identified, but at subsequent surgical excision, no additional tumor is identified. Additionally, this field will identify re-excisions performed for close or positive margins where no additional tumor is identified following the re-excision.

If field 2.2 response is "unknown malignant diagnosis" and this is the initial identification of breast cancer, select "Yes".

5.2	***Field***:	Maximal pathological tumor size
	Response options:	Numeric “xxx.x”
	Instructions:	Record tumor size in millimeters “xxx.x” (mm), for this specific breast excision; if no tumor size recorded, enter -99
	Location:	Pathology report<br><br>If Field 5.1 Residual Tumor = “No” and this is the first surgical excision, then refer to initial pathology report of diagnostic core biopsy for maximal pathologic tumor size, if a size is recorded for core biopsy. If no size for core biopsy is recorded, enter -99.<br><br>If Field 5.1 Residual Tumor = “No” and this is a surgical re-excision following partial mastectomy, then record -99.
	Definition:	This is the largest reported size of tumor (maximal contiguous focus) identified on the pathology report for this operation only. If this is the only breast surgery, this is often the final pathologic tumor size (T) used for TNM staging.<br>If both invasive and in situ (DCIS) reported, record the size of largest invasive component only.<br>If both invasive and in situ (DCIS) sizes are reported, please record DCIS size in Field 5.3 below “Maximal Extent of DCIS”.<br>If size is reported only as ‘microinvasive’, record as 0.9 mm.<br>If no tumor size is recorded on any pathology report, AND not listed as “microinvasive” then record value as (-99)

5.3 ***Field:*** Maximal Extent of DCIS

Response options: Numeric “xxx.x”

Instructions: Record in millimeters “xxx.x” (mm). Complete for patients with either IDC and DCIS where the DCIS disease size is recorded **or** for patients with discontiguous DCIS where pathologist specifically indicates a broader extent of DCIS than the maximal pathologic tumor size (recorded in field above)

All else enter “-99”.

Location: Pathology report

Definition: This field is for patients with pathology reports with identified DCIS. Often the total extent of DCIS can be greater than any single measured foci of disease, because of discontiguous disease, or skipped lesions. While the maximal single focus of DCIS may be recorded in field 5.2, any maximal extent of DCIS identified in the pathology report would be recorded in field 5.3.

5.4 ***Field:*** Pathological tumor type

Response Options: DCIS

IDC

ILC

No Residual Tumor

Instructions: Select one value

Location: Pathology Report

Definition: This is the pathologic tumor type identified as the dominant or most concerning histology identified specific to this surgical event. This is often the same as the initial diagnosis. However, it is not uncommon for a pre-op diagnosis of DCIS to prove to be invasive cancer on final pathology, and that pathologic tumor type would be identified here. In situation where there are multiple histology's listed (IDC and ILC), select what appears to be the more prevalent tumor type. If unclear which histology is more prevalent, then select IDC. When invasive and non-invasive are listed as both present, select the invasive component as the pathological tumor type.

DCIS = Ductal Carcinoma in situ

IDC = Infiltrating Ductal Cancer

ILC = Lobular Cancer

Special Instructions:

If field "5.1 Residual Tumor" = No, then select "No Residual Tumor" for 5.4

5.5 ***Field***: Synchronous second tumor, pathologic

Response options: No

Yes – DCIS

Yes – IDC

Yes - ILC

Instructions: Select one value

Location: Pathology report

Definition: Synchronous tumor: A synchronous tumor is defined for this study as a separate, distinct focus of a breast tumor in the same breast, and the same diagnostic event, and most often identified in a single surgical event. Usually, there is a clear area of normal breast tissue between the two foci of identified cancer. It is “simultaneous” to the incident breast cancer, in contradistinction to a “metachronous” tumor, which may be identified in the same breast, but is usually defined as >1 year from the incident breast cancer. NOTE: the co-existence of DCIS and IDC is generally NOT a situation of a synchronous second tumor, as the non-invasive component is generally immediately adjacent to the invasive component. We are specifically seeking to identify situations where there is noted normal breast tissue between two completely separate foci of breast cancer.

If a second breast cancer is identified in the *opposite* breast within a month of the incident cancer, this is second primary, NOT a synchronous tumor.

*For purposes of this study, a synchronous tumor is used to refer to the identification on the pathology report of a distinct tumor.

Synchronous breast tumor size is often NOT recorded in tumor registry data as the tumor is staged by the largest distinct focus of cancer.

Select ‘no’ if there is not another distinct focus of identified breast cancer. If there is a second focus distinct from the original tumor, please select the appropriate histology.

Select ‘no’ for Breast Re-excisions, when residual disease is identified at or near a previous margin as this is not synchronous tumor. For the vast majority of re-excisions, the selection here will be ‘no’. However, if the second surgery is a mastectomy, it is feasible that a second synchronous tumor can be identified.

5.6	***Field:***	Synchronous second tumor size
	Response options:	Millimeters (mm) “xxx.x”
	Instructions:	Complete if field “synchronous second tumor” = “yes”; all else enter “-99”<br>Enter a numeric value “xxx.x” (size in mm)
	Location:	Pathology report
	Definition:	Largest reported size of synchronous tumor (maximal contiguous focus)<br>If the synchronous tumor is reported as ‘microinvasive’, record as 0.9 mm***.***<br>If no tumor size is recorded on any pathology report, AND not listed as “microinvasive” then record value as (-99)

6 Tumor Histologic/Prognostic Features

This data pertains to the synoptic report, summarizing features of patients' breast cancer from all biopsies and surgeries pertinent to this diagnostic event. It is not expected that the information would change from one surgery to the next for the same diagnostic event. For database purposes, it is required that the fields be completed for each surgery, even if the responses are the same.

6.1 ***Field:*** ER Status

Response options:
- Positive
- Negative
- Unknown
- Missing

Instructions: Select one response

Location: Pathology report/Lab report

Definition: Positive value is 1+, 2+, 3+ or ≥1%.

Cases formerly labeled as "borderline" will now be categorized as positive based on likelihood borderline represents >1%.

Negative: ER status testing interpreted as negative

Unknown: Includes "tests not done", "unknown" or "no Information". Unknown includes categories where there is no indication that ER hormone reception was ever completed.

Missing: Ordered but results not available

6.2 ***Field:*** PR Status

Response options: Positive

Negative

Unknown

Missing

Instructions: Select one response ***Location:*** Pathology report/Lab

report ***Definition:*** Positive value is 1+, 2+, 3+ or ≥1%.

Cases formerly labeled as “borderline” will now be categorized as positive based on likelihood borderline represents >1%.

Negative: ER status testing interpreted as negative

Unknown: Includes “tests not done”, “unknown” or “no Information”. Unknown includes categories where there is no indication that ER hormone reception was ever completed.

Missing: Ordered but results not available

**6.3** ***Field:***	Grade of Primary Invasive Breast Tumor
Response Options:	Low<br>Intermediate<br>High<br>Not Recorded<br>Not Applicable
Instructions:	Select one value
Location:	Pathology Report
Definition:	If Pathology Report indicates grade 1 or low grade, select "Low". If report indicates grade 2, moderate or intermediate; select "Intermediate". If report indicates grade 3 or high, select "High".<br>If a pathology grade range is given, select the higher number, e.g. grade I/II, select II or intermediate; grade II/III, select high grade.<br>If invasive cancer and no grade of tumor described, select "not recorded".<br>If final pathology = DCIS, then select "Not Applicable".<br>If core biopsy and surgical excisional biopsy differ on grade, select grade of excisional biopsy.<br>If grade only described on core biopsy, or no additional tumor identified at surgical excision, then select grade as identified on core biopsies.

6.4	***Field:***	Grade of DCIS
	Response Options:	Low<br>Intermediate<br>High<br>Not Recorded<br>Not Applicable
	Instructions:	Select one value
	Location:	Pathology Report
	Definition:	If Pathology Report indicates grade 1 or low grade, select "Low". If report indicates grade 2, moderate or intermediate; select "Intermediate". If report indicates grade 3 or high, select "High".<br>If DCIS is identified in pathology report but grade is not described, then select "not recorded".<br>If invasive cancer only and no concurrent DCIS described, select "Not Applicable"<br>If core biopsy and surgical excisional biopsy differ on grade, select grade of excisional biopsy.<br>If grade only described on core biopsy, or no additional tumor identified at surgical excision, then select grade as identified on core biopsies.

6.5 ***Field***: Lymphovascular invasion present

Response options: Yes

No

Not Recorded

Not Applicable

Instructions: Select one response

Location: Pathology report

Definition: If lymphovascular invasion is present, select 'yes'. If it is not present, select 'no'.

If lymphatic invasion alone is present, select "yes". If vascular invasion alone is present, select "yes". Any mention of significant or identified invasion of lymphatic vessels or blood vessels, or tumor seen in these structures, select "yes".

If no mention of lymphovascular invasion and patient had an invasive cancer (invasive ductal or invasive lobular) select "Not Recorded".

If DCIS only, select "Not Applicable"

6.6 ***Field***: Perineural invasion present

Response options: Yes

No

Not Recorded

Not Applicable

Instructions: Select one response

Location: Pathology report

Definition: If perineural invasion is present, select 'yes'. If it is not present, select 'no'.

If no mention of perineural invasion and patient has an invasive cancer (invasive ductal or invasive lobular) select "Not Recorded".

If DCIS only, select "Not Applicable"

6.7 ***Field:*** Her2/neu value by IHC

Response options: 0

1+

2+

3+

Not Recorded

Not Applicable

Instructions: Select one response

Location: Pathology report/Lab report

Definition: Enter the value of this lab. If listed as "strongly positive", or "diffusely positive", then select 3+.

IHC=Immunohisto chemistry

If no record of Her2/neu testing by IHC being done, select "Not Recorded"

If DCIS only, select "Not Applicable".

**6.8**	***Field:***	Her2/neu value by FISH Not
	Response options:	amplified (negative)
		Equivocal/Amplified (positive)
		Not Recorded
		Not Applicable
	Instructions:	Select one response
	Location:	Pathology report/Lab report
	Definition:	Enter a value of FISH if performed. This test might be performed if Her2/neu by IHC is 2+. This test is generally not required if Her2/neu IHC is either negative or strongly positive.<br>If FISH testing was not performed, or no evidence of result, select "Not Recorded"<br>If DCIS only, select "Not Applicable"

**6.9**	***Field:***	Contralateral prophylactic mastectomy pathology
	Response options:	No incidental cancer
		Incidental invasive cancer
		Incidental DCIS
		Incidental ADH, ALH or LCIS
		Not Applicable
	Instructions:	Complete for patients electing to undergo prophylactic contralateral mastectomy; all else select "Not Applicable".<br>Select one response
	Location:	Pathology report
	Definition:	If a prophylactic mastectomy performed on the contralateral breast, there may be cancer related histologies identified. If any of the above listed histologies are identified please record.

7 Lymph Node Surgery (s)

Each distinct lymph node surgery done for a single diagnosis date for each individual breast requires this section to be completed for each procedure. For purposes of this study, we will consider SLN surgery and axillary dissection as distinct procedures, even if completed on the same date under the same anesthetic. For instance, if a patient first underwent SLN surgery, followed by axillary dissection, this section would be completed x 2, regardless of whether the same procedure date or different procedure dates.

If no lymph node surgery ever performed:
Paper data collection tool: Select "none" for field 7.4 Axillary Procedure Type and leave all else blank.
Electronic data entry: None of these fields should appear.

7.1 ***Field:*** Date of Lymph Node Surgery
Response Options: Date
Instructions: Record date of procedure – mm/dd/yyyy
Location: Surgeon operative report
Definition: Record Date of lymph node surgery. Lymph node surgery is defined as either sentinel node biopsy, or axillary dissection or axillary sampling.

7.2 ***Field:*** Surgeon ID
Response Options: List and crosswalk is available at your site.
Instructions: Select one value
Location: Should be pre-populated; otherwise surgeon's report
Definition: Unique random number assigned by the programmer who will keep a crosswalk to the internal physician identifier that will not be shared beyond the individual site. This should be the surgeon who conducted the initial breast cancer surgery.

7.3 ***Field:*** Facility
Response Options: List and crosswalk is available at your site.
Instructions: Select one value
Location: Medical Record
Definition: Name of the facility/hospital/surgical clinic where the initial surgery was done.

7.4	***Field:***	Axillary Procedure Type
	Response Options:	None
		SLN – successful identification
		SLN – failed identification
		Axillary Dissection
		Axillary sampling
	Instructions:	Select one value
	Location:	Surgeon operative report
	Definition:	None: Select this option if no axillary procedure was ever performed as a component of treatment or staging for this incident breast cancer diagnosis.<br>Criteria for identifying a sentinel node includes a 'hot' node detected by gamma probe, a blue lymph node or lymph node with a blue stained lymphatic leading into it, and any grossly palpable or otherwise suspicious/abnormal lymph node found intra-operatively<br>SLN-Successful identification: surgeon's operative report indicates that a SLN procedure was performed and a node was identified (often hot or blue AND the pathology report indicates a node was identified).<br>SLN: Failed identification: The surgeon cannot identify anything in the operating room as a sentinel node. Operative report may indicate failure to identify a "hot spot" or any "hot" or "blue node". Also, it is possible for the surgeon to remove a packet of tissue, believing a node to have been identified, but no nodes are identified in the tissue when analyzed by the pathologist. In that case, select "SLN-failed identification"<br>Axillary Dissection (AD): this is generally an anatomic dissection where the borders of the pectoralis major muscle are identified, the latissimus dorsi is identified, and the axillary vein is identified. Almost always the surgeon describes some effort to identify motor nerve within the axilla. This is the more traditional method of removing lymph nodes for breast cancer, and is now often performed just selectively following a SLN biopsy where a positive SLN is identified.<br>Axillary sampling (AS): A sampling of soft tissue in the axilla is taken without formally identifying anatomic structures of the axilla. Select AS if an axillary nodal excisional biopsy was performed for diagnosis of breast cancer (in cases of nodal metastasis in absence of primary). May be performed when SLN cannot be identified. Generally no mention of identifying the axillary vein or nerves when sampling only is performed.<br><br>SPECIAL INSTRUCTION:

If axillary dissection (AD) or axillary sampling (AS) was performed subsequent (either immediately or at a later date) to the SLN procedure, then record AD or AS as a second lymph node procedure with the appropriate date (same date as SLN or subsequent date). In some instances when Neoadjuvant therapy is utilized, some centers perform the SLN biopsy before neoadjuvant treatment is initiated.

7.5 ***Field:*** Intra-operative SLN pathologic evaluation

Response Options: No

Yes – positive

Yes – negative

Yes – Not Recorded

Not Applicable

Instructions: Select one

Location: Surgeon's operative note and pathology report

Definition: This involves the SLN being sent to pathology for an intra-operative interpretation.

If no intra-operative assessment on the SLN was performed select "No".

If an intra-operative pathologic assessment of the sentinel node was performed at the time of the SLN procedure please select Yes-positive (if pathologist interpreted the node to be positive),

Yes-negative (if the pathologist interpreted the node to be negative), or

Yes-Not Recorded (if an intra-operative assessment was performed but the determination of the nodal status intra-operatively is not recorded).

Select "Not Applicable" if no SLN procedure was performed.

7.6 ***Field:*** SLN Histology

Response Options:
Negative
Positive
Positive – Micrometastasis only
Positive – ITC only
Not Applicable

Instructions: Select one value

Location: Pathology Report - - From final surgical pathology report. This selection does not represent the intra-operative interpretation of SLN if an intra-operative analysis was performed.

Definition: Positive: Select this option if tumor identified by standard pathology H&E staining.

Positive-Micrometastasis only is defined as tumor deposits between 0.2 and 2 mm found within the lymph node.

Positive-Isolated tumor cells (ITC) is defined as the presence of tumor cells or clusters measuring less than 0.2 mm found within the lymph node.

Select "Not Applicable" if no SLN procedure was performed.

**7.7**	***Field:***	SLN Positive Node Count
	Response Options:	Numeric
	Instructions:	Whole integer. Enter number of positive sentinel nodes; if no positive nodes identified, enter 0.<br>If only isolated tumor cells (ITC) reported by pathology in the SLN, enter 0.<br>If no SLN surgery performed, enter -99. If procedure is a subsequent axillary dissection, enter -99.
	Location:	Pathology report
	Definition:	This number should represent the number of SLN that were identified to be involved with breast cancer metastases.<br>Do include micrometastases in the positive SLN count.<br>Do not include ITC only in the positive sentinel node count.<br>Criteria for identifying a sentinel node includes a 'hot' node detected by gamma probe, a blue lymph node or lymph node with a blue stained lymphatic leading into it, and any grossly palpable or otherwise suspicious/abnormal lymph node found intra-operatively

**7.8**	***Field:***	SLN Total Node Count
	Response Options:	Numeric
	Instructions:	Whole integer. Enter total number of sentinel nodes.<br>If no SLN surgery was performed enter "-99". If procedure is a subsequent axillary dissection, enter -99.
	Location:	Pathology report
	Definition:	This number should represent the total number of sentinel nodes identified from the sentinel node procedure only. Typically the range here is 1-5. The number is taken from the pathology report. It is not uncommon for the surgeon to believe they have removed just a single node and then have the pathologist identifies 2-3 nodes in the packet of tissue removed by the surgeon.

7.9 ***Field:*** AD Positive Node Count

Response Options: Numeric

Instructions: Whole integer. Enter number of positive nodes identified on AD or axillary sampling (AS). Include intramammary or other nodes identified in final path report. Do NOT include the positive SLN in this count, if a SLN procedure performed. If no nodes are positive, enter zero.

If no AD or AS was performed, enter -99.

Location: Pathology report

Definition: This number should represent the additional number of lymph nodes from the AD or AS that were identified to be involved with breast cancer metastases.

Do NOT include the positive SLN total count in this number.

7.10 ***Field:*** AD Total Node Count

Response Options: Numeric

Instructions: Whole integer. This number should represent the additional number of lymph nodes identified from the AD or AS. Enter total number of AD nodes.

If no AD or axillary sampling was performed, enter "-99".

Do NOT include the SLN total count in this number.

Location: Pathology report

Definition: This number represents the total number of lymph nodes identified by the pathologist following the AD or axillary sampling. e.g., if a total of 20 nodes were examined between the SLN and the AD, and three were sentinel nodes, then this number would be 17.

7.11 ***Field:*** Record Complete

Response Options: Yes

N
o

Instructions: Select one value

Location:

Definition: Yes = Select only if **<u>all</u>** fields and data elements are complete. Regardless if they are to be electronically imported or entered by the abstractor.

No = At least one field or data element is still missing for the patient.

List of Abbreviations

A = anterior (toward the skin overlying the breast tissue) AD =

Axillary lymph node dissection

ADH = Atypical ductal hyperplasia ALD =

Axillary lymph node dissection AS =

Axillary sampling

DCIS = Ductal Carcinoma In Situ, also known as intraductal breast cancer

ER = Estrogen receptor

FISH = Fluorescence in situ hybridization

FNA = Fine Needle Aspirate

HER2/neu = Human epidermal growth factor receptor 2

I = inferior (toward the patient's feet) IDC

= Invasive ductal cancer

IHC = Immunohistochemistry ILC =

Invasive lobular cancer ITC =

Isolated Tumor Cells

L = lateral (toward the axilla/arm) LCIS

= Lobular carcinoma in-situ LN =

Lymph Node

M= medial (toward the sternum)

mm = millimeters

MRM= modified radical mastectomy (total mastectomy + axillary nodal dissection)

P = posterior (toward the chest wall); sometimes recorded as "deep" margin

PM = Partial mastectomy (also known as lumpectomy) PR =

Progesterone receptor

S = superior (toward the patient's head) SLN

= Sentinel lymph node

TM = Total Mastectomy

U/S = Ultrasound
